# The role of STIM and ORAI proteins in phagocytic immune cells

**DOI:** 10.1152/ajpcell.00360.2015

**Published:** 2016-04-01

**Authors:** Nicolas Demaurex, Paula Nunes

**Affiliations:** Department of Cell Physiology and Metabolism, University of Geneva, Geneva, Switzerland

**Keywords:** calcium, phagocytosis, phagocytes, STIM1, ORAI1, store-operated calcium entry

## Abstract

Phagocytic cells, such as neutrophils, macrophages, and dendritic cells, migrate to sites of infection or damage and are integral to innate immunity through two main mechanisms. The first is to directly neutralize foreign agents and damaged or infected cells by secreting toxic substances or ingesting them through phagocytosis. The second is to alert the adaptive immune system through the secretion of cytokines and the presentation of the ingested materials as antigens, inducing T cell maturation into helper, cytotoxic, or regulatory phenotypes. While calcium signaling has been implicated in numerous phagocyte functions, including differentiation, maturation, migration, secretion, and phagocytosis, the molecular components that mediate these Ca^2+^ signals have been elusive. The discovery of the STIM and ORAI proteins has allowed researchers to begin clarifying the mechanisms and physiological impact of store-operated Ca^2+^ entry, the major pathway for generating calcium signals in innate immune cells. Here, we review evidence from cell lines and mouse models linking STIM and ORAI proteins to the control of specific innate immune functions of neutrophils, macrophages, and dendritic cells.

## A Brief Primer on Store-Operated Ca^2+^ Entry

ca^2+^ is a ubiquitous second messenger important for a large variety of cellular functions. Intracellular Ca^2+^ signaling is based on the principle that cytosolic Ca^2+^ concentrations are maintained at levels ∼100,000-fold lower than the extracellular environment by energy-driven pumps that either extrude Ca^2+^ ions from the cell, or sequester them in membrane-bound intracellular compartments, termed Ca^2+^ stores. This large concentration gradient then allows an extremely rapid influx of Ca^2+^ upon regulated opening of Ca^2+^ channels, either at the plasma membrane (PM) or on the membranes of Ca^2+^ stores. The major intracellular store is the endoplasmic reticulum (ER), where pumps of the sarcoendoplasmic-reticulum ATPase family maintain luminal Ca^2+^ levels high under steady-state conditions, although the role of lysosomes as Ca^2+^ storage and signaling platforms has been increasingly recognized more recently (84). In excitable cells, such as muscles and neurons, the opening of voltage-gated Ca^2+^ channels upon membrane depolarization is one of the principle manners by which cytosolic Ca^2+^ signals are generated, in addition to the direct opening of ligand-gated Ca^2+^ channels (4, 11). In contrast, in nonexcitable cells, comprising most other cell types and including cells of the immune system, membrane depolarization alone is not sufficient to generate cytosolic Ca^2+^ signals, and, instead, Ca^2+^ signaling in these cells is generally considered to be dominated by the mechanism termed store-operated Ca^2+^ entry (SOCE). As the name implies, SOCE has been functionally defined as the opening of PM Ca^2+^ channels that occurs subsequent to and as a consequence of the release of Ca^2+^ from intracellular stores, and was historically also termed capacitive Ca^2+^ entry (91) and associated with the prototypical Ca^2+^-release activated current (CRAC). Although for decades the CRAC current could be identified through its unique electrophysiological properties, the molecular machinery underlying SOCE was not described until 2005–2006, with the characterization of STIM and ORAI proteins (32, 62, 85, 96, 107, 127).

Under physiological conditions, SOCE is initiated by activation of cell surface or intracellular receptors that trigger the release of Ca^2+^ from the ER. This usually occurs following G protein-coupled or tyrosine-coupled receptor engagement that leads to the activation of phospholipase C, which in turn cleaves the membrane phospholipid phosphatidylinositol (4,5)-bisphosphate generating diacyl glycerol and inositol trisphosphate (InsP_3_). InsP_3_ then diffuses from the site of activation to the surface of the ER, where it binds InsP_3_ receptors that allow Ca^2+^ to flow out of the ER and into the cytosol (91). Other mechanisms of Ca^2+^ release from the ER exist, via the activation of ryanodine receptors, whose expression is most prominent in muscle cells, or via phospholipase D-mediated activation of sphingosine kinase and subsequent sphingosine-1-phosphate-dependent ER Ca^2+^ release through an unknown channel (55, 75). Passive Ca^2+^ release from the ER can also occur following physiological or pharmacological inhibition of sarcoendoplasmic-reticulum ATPase pumps due to nonspecific leakage through the translocon Sec61 complex (53) and potentially other unknown ER channels or transporters.

STIM proteins are single-pass transmembrane proteins that reside on the membrane of the ER and possess a luminal Ca^2+^ sensing domain composed of two Ca^2+^-binding EF-hand motifs. When ER Ca^2+^ becomes low due to Ca^2+^ release or leak, the dissociation of Ca^2+^ ions from these EF-hands induces a conformational change in the protein that promotes oligomerization and unfolding of the cytosolic domain, exposing additional sites for protein-protein interactions. STIM protein activation is also accompanied by its microtubule-driven translocation along the ER membrane to sites of tight apposition or contact between the ER and the PM. There, its lysine-rich COOH-terminal tail can interact directly with PM phospholipids, strengthening and stabilizing the ER-PM contacts, and the exposed CRAC activation domain/STIM ORAI activation region (CAD/SOAR) activation domains can trap and directly gate PM Ca^2+^ channels. In addition to luminal ER Ca^2+^ content, the activity of STIM proteins are regulated by phosphorylation, ROS-dependent cysteine modification, as well as binding to regulatory proteins, including junctate (ASPH), partner of stromal interaction molecule 1 (POST/TMEM20/SLC35G1) and store-operated calcium entry associated factor (SARAF/TMEM66) (reviewed in Refs. 90, 106).

ORAI proteins are the principle type of PM Ca^2+^ channels to be gated by STIM proteins. They are highly selective for Ca^2+^ and are composed of four transmembrane domains, with both NH_2_- and COOH-termini facing the cytosol. They operate as tetramers or hexamers and interact with STIM proteins with both luminal domains. They are also regulated by phosphorylation and ROS-dependent cysteine modifications, as well as binding to regulatory proteins, such as CRAC regulators (CRACR2A/EFCAB4B and CRACR2B/EFCAB4A), and additionally by their translocation to phosphatidylinositol (4,5)-bisphosphate-rich microdomains of the PM (12, 42, 63, 74). STIM proteins additionally modulate the activity of nonselective cation channels of the transient receptor potential (TRP) cation family, but whether this interaction is direct or occurs via ORAI proteins is still under debate (81, 123, 125).

There are three known members of the STIM family of proteins: STIM1, STIM2, and STIM1L, which have differing Ca^2+^ sensitivities and functions. While STIM1 and STIM2 both display a broad tissue distribution, STIM1L isoforms, a product of alternative splicing of the STIM1 gene, are more prominently expressed in muscle cells. STIM1 is by and large the principle master regulator of SOCE in most cell types tested (with the exception of neurons), while the function of STIM2 is less understood. STIM2 has a higher sensitivity for Ca^2+^ and thus becomes activated before STIM1, but is less potent as a ligand for ORAI channels, and its highly conserved alternatively spliced isoform STIM2β is a potent inhibitor of SOCE (92). STIM2 has been suggested to control basal cytosolic and ER Ca^2+^ levels and might play a more important role at low levels of receptor activation. Similarly there are three known isoforms of ORAI channels: ORAI1, ORAI2 and ORAI3, all of which are broadly expressed with differing expression levels, depending on cell type. While ORAI1 appears to be the main isoform to partner with STIM1, ORAI3 has been shown to be more resistant to ROS than ORAI1, and, although it has a lower Ca^2+^ conductivity than ORAI1, its upregulation may represent an important adaptation to oxidative stress (12, 74). In addition, ORAI3 and ORAI1 form heteromultimers that give rise to a different type of channel, the arachidonate-regulated or leukotriene-regulated channel (ARC and LRC, respectively), involved in an alternative form of Ca^2+^ entry termed *I*_arc_ in certain cell types, including parotid and pancreatic acinar, vascular smooth muscle, and taste bud cells under physiological conditions, and cardiomyocytes and certain types of cancer cells under pathological conditions. Activation of these channels is independent of store depletion and instead relies on the direct binding of arachidonic acid or leukotriene C4 to ORAI3, as well as both ER-resident STIM1 and a small pool of STIM1 targeted to the PM (28, 68, 128, 129). ORAI1 has an additional splice variant ORAI1β that lacks an NH_2_-terminal polyarginine domain, is more mobile due to lower binding to PM phosphoinositides, has lower Ca^2+^-dependent inhibition, and does not participate in arachidonate-regulated or leukotriene-regulated channel formation (22, 36). The function of ORAI2 is less understood.

The importance of STIM and ORAI function in immunity is highlighted by the fact that loss of function mutations of either protein in humans is characterized by severe immunodeficiency (32, 88, 100) (reviewed in Refs. 33, 52). Although extensive work has shown the importance of T-cell dysregulation as a major factor in this immunodeficiency, certain aspects like recurrent mycobacterial infections may also be consistent with defects in innate immune cells. The manipulation of extracellular and intracellular Ca^2+^ using chelators and the discovery of pharmacological inhibitors capable of inhibiting CRAC currents has allowed a long history of research highlighting the importance of Ca^2+^ signals in phagocytes for a variety of different functions and hinting to the importance of STIM and ORAI in phagocytic immune cells. However, the literature is also littered with inconsistencies and controversies (discussed in Refs. 75, 104). Part of this may be due to the fact that many inhibitors, such as 2-aminoethoxydiphenyl borate and SKF-96365 have pleiotropic effects beyond their effects on CRAC channels. However, another reason likely stems from the differences in experimental models and conditions, since primary phagocytic cells can be short-lived and are generally difficult to transfect or transduce, and, although some acceptable cellular models exist, they seldom recapitulate phagocyte function to their full extent. This difficulty in experimental manipulation may also explain why after nearly 10 yr since their discovery, the function of STIM and ORAI in phagocytes is only now beginning to be understood, particularly as knockout mouse models are becoming increasingly exploited. In the following sections, we review recent advances in the understanding of SOCE in phagocytes, focusing on studies that have directly manipulated STIM and ORAI function in neutrophils, macrophages, and dendritic cells (DCs).

## Neutrophils

Neutrophils are the most abundant white blood cells and our first line of defense against bacterial and fungal infections. Neutrophils sense danger signals via pattern recognition receptors (PRRs) and pathogen- or danger-associated molecular pattern (PAMP and DAMP) receptors and migrate across the vascular endothelium toward infection sites to ingest and kill invading pathogens. Neutrophil emigration across inflamed endothelium is a multistep process initiated by the engagement of selectin and chemokine receptors that trigger a transition from cell rolling to integrin-dependent arrest and migration (reviewed in Ref. 59). Once arrived at the site of infection or injury, neutrophils ingest foreign particles and degrade them within phagocytic vacuoles that gain enzymatic and oxidative properties through fusion with secretory granules containing bactericidal enzymes and a superoxide-generating NADPH oxidase (49). Neutrophils can also eliminate extracellular pathogens by generating superoxide or releasing granules in the extracellular space, as well as releasing neutrophil extracellular traps (NETs) made of chromatin DNA bound to histones and granular proteins (16). Several effector functions of neutrophils were shown to be modulated by either global or local elevations in cytosolic Ca^2+^ concentration. Early studies revealed that Ca^2+^ elevations contribute to the coordinated selectin, chemokine, and integrin signaling cascades that control neutrophil adhesion, spreading, and migration (47, 51, 72, 86, 87). Ca^2+^ elevations direct the remodeling of the actin cytoskeleton (9, 27), as well as integrin recycling and uropod retraction during neutrophil migration (29, 56). Ca^2+^ elevations finely regulate the exocytosis of the different neutrophil granule populations (58, 77) and the production of superoxide by NADPH oxidase located in the PM and in phagosomes (35, 38, 111) (reviewed in Ref. 15). Ca^2+^ elevations also regulate the efficiency of phagocytosis, an effect that likely reflects the Ca^2+^ dependency of the maturation process (46, 121), as the phagocytic ingestion phase appears to be largely Ca^2+^ independent in neutrophils, depending on the type of phagocytic receptors engaged (reviewed in Ref. 75). Although the evidence for Ca^2+^ regulation of neutrophil functions is overwhelming, one major caveat is that most studies relied either on nonspecific inhibitors such as 2-aminoethoxydiphenyl borate or SKF-96365 to target Ca^2+^ release or Ca^2+^ entry channels, or on deprivation of extracellular Ca^2+^, a procedure that quickly depletes intracellular Ca^2+^ stores and interferes with adhesion and migration assays by preventing neutrophil capture by selectin. The identification of the CRAC channel component ORAI1 and its activating intracellular ligand STIM1 in 2005–2006 has now enabled researchers to address the role of Ca^2+^ entry pathways at the molecular level, both in cell lines and in animal models. The studies focusing on neutrophils, some of which were reviewed recently (18), are summarized below and compiled in Tables 1 and 2.

**Table 1.** Neutrophil defects in mouse models of STIM and ORAI deficiency

Mouse Neutrophils		In Vitro			In Vivo		
Gene	Mouse model	Ca^2+^ signaling	Adhesion/migration	Phagocytosis/ROS	Neutrophil recruitment	Pathology	Ref. No.
*Stim1*	*Stim1*^*−/−*^ bone marrow chimera		Normal chemotaxis (C5a, MIP-2)		Normal (lung, IgG-IC)		13
	LysM-Cre myeloid ablation	Decreased (local Ca^2+^ signals)		Decreased phagocytosis (opsonized RBC)			73
	*Stim1*^*−/−*^ fetal liver chimera	Decreased (fMLP, MIP-2, IC, pRGD)	Normal adhesion (fibrinogen); normal chemotaxis (MIP-2, fMLP)	Decreased phagocytosis (*S. aureus*); decreased ROS production (fMLP, pRGD, zymosan)	Normal (Thy peritonitis)	Increased bacterial pneumonia & septicemia; decreased liver ischemia-reperfusion injury	126
	LysM-Cre myeloid ablation	Decreased (Tg)	Reduced chemotaxis (fMIVIL, WKY, KC, MIP-2)		Decreased (skin, IMQ)	Faster recovery of psoriasis-like plaques	112
	*Stim1*^*−/−*^ bone marrow chimera	Decreased (Tg) Normal (C5a, fMLP)	Normal chemotaxis (fMLP)				109
*Orai1*	Heterozygous *Orai*^*+/−*^ mice	Decreased (Tg) Decreased (fMLP)	Delayed polarization (fMLP, Tg/Ca^2+^); delayed arrest (fMLP, Tg/Ca^2+^)				101
	Heterozygous *Orai*^*+/−*^ mice	Decreased (fMLP, ICAM-1 in shear flow)	Reduced adhesion strengthening under flow (ICAM-1); reduced directional migration		Decreased (skin wounds)		25, 26
	*Orai1*^*−/−*^ bone marrow chimera	Normal (Tg) Decreased (C5a, fMLP)	Reduced chemotaxis (C5a, fMLP, LPS-primed biological fluids)		Decreased (C5a peritonitis)		109

The table lists the neutrophil defects reported in mouse models of *Stim1* and *Orai1* deficiency. LysM-Cre, knock-in allele with Cre recombinase inserted into the lysozyme 2 gene; Tg, thapsigargin; IC, immune complexes; MIP-2, macrophage inflammatory protein-2; ICAM-1, intercellular adhesion molecule-1; RBC, red blood cells; Thy, thioglycollate; IMQ; imiquimod; fMLP, *N*-formyl-methionyl-leucyl-phenylalanine.

**Table 2.** Neutrophil defects in human cell lines and in STIM1 and ORAI1 deficient patients

Gene	Manipulation	Ca^2+^ Signaling	Adhesion/Migration	ROS Production	Ref. No.
HL-60 cells					
*STIM1*	siSTIM1	Decreased (Tg, fMLP)		Decreased (Amplex Red, fMLP)	14
		Decreased (FcγR)		Decreased (DCFH2-yeast)	113
		Decreased (Tg, fMLP)	Decreased polarization (fMLP)		131
			Reduced chemotaxis (fMLP)		112
*STIM2*	siSTIM2	Normal		Normal	14
*ORAI1*	siORAI1	Decreased (Tg, fMLP)	Delayed arrest and polarization (fMLP, Tg/Ca^2+^)		101
		Decreased (FcγR)		Decreased (DCFH2-yeast)	113
			Decreased adhesion strengthening to ICAM-1/E-selectin under flow		25
			Abrogated chemotaxis (fMLP)		112
*ORAI2*	siORAI2	Normal			
*ORAI3*	siORAI3	Normal			
*STIM1*	YFP-STIM1 overexpression	Increased (fMLP)	Increased chemotaxis (fMLP)		112

Gene	Mutation	Ca^2+^ Signaling	Adhesion/Migration	Phagocytosis/ROS	Cytokine Secretion	
Human neutrophils						
*STIM1*	p.R429C	Normal (fMLP, streptococci)	Normal (fMLP, PAF, C5a)	Normal (streptococci, zymosan)	Normal (IL-8)	(30)
*ORAI1*	p.R91W	Normal (Tg, fMLP, streptococci)	Normal (fMLP, PAF, C5a)	Normal (streptococci, zymosan)	Normal (IL-8)	(30)

The table lists the in vitro defects reported in neutrophil-like HL-60 cells following silencing and overexpression of *STIM* and *ORAI* isoforms, and in patients lacking functional STIM1 and ORAI1. DCFH2, 2′,7′- dichlorodihydrofluorescein; FcγR, immunoglobulin-γFc region receptor.

### Cell lines.

The most studied neutrophil cellular model is the myelomonocytic cell line HL-60, which can be driven toward a neutrophil-like phenotype upon differentiation with DMSO. Several studies reported the effect of STIM or ORAI silencing in DMSO-differentiated HL-60 cells (Table 2). In all cases, the reduction in protein levels ranged between 50 and 70%. The first study reported that STIM1 but not STIM2 silencing reduced by 50% the extracellular production of H_2_O_2_ measured by Amplex Red (14). In a subsequent study, the same group reported that STIM1 and ORAI1 silencing, but not ORAI2 and ORAI3 silencing, abrogated the global cytosolic Ca^2+^ elevations evoked by the ligation of FcγR during phagocytosis of opsonized yeast particles. In this case, the intraphagosomal production of ROS measured with DCFH2-labeled particles was reduced by ∼50% (113). STIM1 silencing also decreased the polarization of HL-60 cells exposed to the chemotactic peptide *N*-formyl-methionyl-leucyl-phenylalanine (fMLP) by 70%, an effect that correlated with a decreased phosphorylation of Akt, Src, and Rac2 (131). Similarly, ORAI1 silencing delayed the onset of arrest of HL-60 cells rolling on E-selectin and reduced their ability to adopt a polarized migratory phenotype upon exposure to fMLP or thapsigargin (101). In a recent study, STIM1 and ORAI1 small interfering RNA silencing reduced chemotaxis to fMLP by 50 and 100%, respectively, while YFP-STIM1 overexpression caused a doubling in the chemotactic index and in the amplitude of the Ca^2+^ response to fMLP (112). Together, these studies indicate that knockdown of STIM1 or ORAI1 has a significant impact on Ca^2+^ elevations, ROS production, and chemotaxis in neutrophil-like HL-60 cells.

### Animal models.

Several studies have used transgenic mice to study the effect of *Stim1*, *Stim2*, or *Orai1* ablation in innate immune cells (Tables 1, 3, and 4). Three studies focused on macrophages (13, 108, 117), and four on neutrophils (73, 109, 112, 126). To overcome the lethality associated with global *Stim1* and *Orai1* deletion, these studies relied either on lineage-specific gene ablation (73, 112, 117); on bone marrow transplantation of fetal cells from knockout mice (13, 108, 109, 126), or on the use of heterozygous *Orai1*^*+/−*^ mice (25, 26, 101). Different genetic backgrounds were used, with myeloid-specific ablation performed in a pure B6 background, while the *Orai1*^*+/−*^ heterozygous mice and the donor knockout mice used to reconstitute the granulocyte compartment in radiation chimera were backcrossed for six generations with outbred mice to improve survivability.

**Table 3.** Macrophage defects in mouse models of STIM and ORAI deficiency

Mouse Macrophages		In Vitro				In Vivo		
Gene	Mouse model	Ca^2+^ signaling	Chemotaxis	Phagocytosis	Cytokine production	Macrophage function	Pathology	Ref. No.
*Stim1*	*Stim1*^*−/−*^ bone marrow chimera	Decreased SOCE and store content (Tg, PM)			Decreased (C5a)	Reduced phagocytosis (Kupfer cells)	Protected from AIHA, ITP, and pneumonitis	13
	*Stim1*^*−/−*^ bone marrow chimera	Decreased (Tg, FcγRIII and RIV, PM)	Normal (C5a, CCL2)	Decreased (PM, opsonized RBC)	Normal (TNF and IL-6 in response to LPS)	Normal cytokine secretion (ip LPS injection). Normal macrophage recruitment (Thy peritonitis)	Reduced IgG-induced hemolytic anemia	108
*Stim1* and *Stim2*	Mx1-Cre or Vav-Cre *Stim1*^*−/−*^; *Stim2*^*−/−*^ myeloid ablation	Decreased SOCE, normal store content (Tg, BMDM)		Normal (BMDM & PM) IgG-mediated. Normal phago-lysosome fusion	Normal (IL-2, IL-6, IL-10, IL-12p40, IL-12p70, IL-23p19, TNF-α in response to LPS, curdlan, BCG), IL-1b in response to ATP, MSU, FlaTox			117
*Stim2*	*Global Stim2*^*−/−*^ and *Stim2*^*−/−*^ chimera	Decreased SOCE and store content (Tg) Decreased (FcγR)	Decreased (C5a, CCL2)	Decreased 20% (PM, opsonized RBC)	Decreased (TNF and IL-6 in response to LPS)	Reduced cytokine TFN, IL-6, IL-1β secretion (ip LPS injection). Reduced macrophage recruitment (Thy peritonitis)	Increased survival following LPS injection	108
*Orai1*	*Orai1*^*−/−*^ chimera	Decreased (Tg, PM)	Normal (C5a, CCL2)			Normal macrophage recruitment (Thy peritonitis)		109

The table lists the macrophage defects reported in mouse models of *Stim1*, *Stim2*, and *Orai1* deficiency. Vav-Cre, knock-in allele with Cre recombinase under control of murine *Vav* gene regulatory elements; BMDM, bone-marrow derived macrophage; PM, peritoneal macrophages; AIHA, autoimmune hemolytic anemia; ITP, idiopathic thrombocytopenia purpura; SOCE, store-operated Ca^2+^ entry; BMDM, bone marrow-derived macrophage; RBC, red blood cell; BCG, Bacille Calmette-Guerin; MSU, monosodium urate; ip, intraperitoneal.

**Table 4.** Macrophage defects in cell lines

RAW 264.7 cells		In Vitro			
Gene	Manipulation	Ca^2+^ signaling	Chemotaxis	Phagocytosis	Ref. No.
*Stim1*	siStim1	Decreased 70% (Tg)	Normal (C5a, CCL2)	Decreased 75% (opsonized RBC)	109
*Stim2*	siStim2	Decreased 30% (Tg). Decreased store content	Reduced 20% (C5a, CCL2)	Decreased 50% (opsonized RBC)	109

The table lists the macrophage defects reported in the mouse monocyte/macrophage cell line RAW 264.7 (Abelson murine leukemia virus transformed) following silencing of *Stim* isoforms.

In a pioneering study, Braun et al. (13) used lethally irradiated mice transplanted with bone marrow cells from *Stim1*^*−/−*^ mice. They reported normal neutrophil migration toward the cytokine macrophage inflammatory protein-2 (MIP-2, also known as CXCL2) or the anaphylatoxin C5a in vitro, and normal neutrophil infiltration into lungs in a model of immune complex-induced pneumonitis. Whether this phenotype correlated with alterations in neutrophil Ca^2+^ signals is unclear, however, as SOCE was not measured in these *Stim1*^*−/−*^ neutrophils. In a subsequent study, we used the myeloid-specific LysM-Cre promotor to delete the *Stim1* gene in neutrophils from C57BL/6 mice (*Stim1*^*fl/fl*^; *LysM*^*Cre/+*^). *Stim1* ablation decreased phagocytosis in neutrophils by 50% and caused a corresponding 50% decrease in the frequency of the local Ca^2+^ elevations occurring near phagosomes (73). To our surprise, the global SOCE response evoked by the thapsigargin/Ca^2+^ readmission protocol was preserved in STIM1-deficient neutrophils (data not shown). Yet *Stim1* deletion decreased the extent and length of cortical ER cisternae juxtaposed to phagosomes and to the PM by 50%. We, therefore, concluded that STIM1 recruits ER cisternae near phagosomes to generate Ca^2+^ microdomains that promote the phagocytic process. The prophagocytic Ca^2+^ signals were generated by the release of Ca^2+^ from the recruited ER stores and by the opening of phagosomal Ca^2+^ channels by STIM1, implying a dual role for STIM1 as an adaptor protein that delivers “calciosomes” to specific cellular locations and as ligand for intracellular channels. The study suggests that local Ca^2+^ signals might be more important than global Ca^2+^ signals in the control of neutrophil function.

More recently, Zhang et al. (126) used irradiated mice transplanted with fetal liver cells from *Stim1*^*−/−*^ mice to study the impact of *Stim1* ablation on neutrophil function. The delayed phase of the Ca^2+^ elevations evoked by chemoattractant, Fcγ-receptor cross-linking, store depletion, and integrin-mediated adhesion were reduced in *Stim1*^*−/−*^ neutrophils. The extent of inhibition was not quantified, but the representative traces suggest a near complete abrogation of Ca^2+^ entry triggered by receptor ligation in *Stim1*^*−/−*^ neutrophils. Surface levels of adhesion molecules LFA-1 (lymphocyte function-associated antigen 1, β_2_-integrin CD11a/CD18) and Mac-1 (CD11b/CD18), adhesion to fibrinogen, PLC-γ phosphorylation, and InsP_3_ production were normal in *Stim1*^*−/−*^ neutrophils, ruling out upstream defects in integrin signaling. Despite the SOCE defect, *Stim1*^*−/−*^ neutrophils efficiently migrated toward chemoattractant in vitro and were recruited in the peritoneal cavity and in foot pads following injection of thioglycollate and MIP-2. Phagocytosis of opsonized *S. aureus* was reduced by 50–70%, and integrin and chemoattractant-mediated lactoferrin release, by 30%. A more profound defect was observed for superoxide production measured with the luminol-based chemiluminescence and cytochrome *c* reduction assays, the inhibition ranging from 25 to 50% for FcγR-mediated to 50–100% for integrin and chemoattractant-mediated responses. The authors concluded that STIM1 is dispensable for neutrophil adhesion and migration, and that the major cellular defects of *Stim1* ablation in neutrophils are a defective respiratory burst, reduced phagocytosis, and a mild degranulation defect. The blunted superoxide production of *Stim1*^*−/−*^ neutrophils was linked to reduced phosphorylation of the p40^*phox*^ and p47^*phox*^ NADPH oxidase subunits by PKC-α and -β. In vivo, mice engrafted with *Stim1*^*−/−*^ bone marrow cells were more susceptible to *S. aureus* pneumonia and *L. monocytogenesis* septicemia and had reduced liver damage following ischemia-reperfusion injury, with levels of liver enzymes and of the inflammatory cytokine IL-6 comparable to those observed in p47^*phox−/−*^ mice lacking a functional oxidase. This rather spectacular phenotype cannot be unambiguously attributed to defective neutrophil function, however, because SOCE deficiency in macrophages and DCs might contribute to the increased susceptibility of *Stim1*^*−/−*^ chimera to bacterial infections, while impaired platelet function might account for the reduced tissue injury upon hepatic ischemia-reperfusion.

In a parallel study, Steinckwich et al. (112) investigated mice bearing a homozygous LysM-Cre driven myeloid-specific ablation of Stim1 (*Stim1*^*fl/fl*^; *LysM*^*Cre/Cre*^). The STIM1-deficient neutrophils isolated from the blood and bone marrow of these mice had ∼50% reduced Ca^2+^ entry following store depletion with thapsigargin and reduced chemotaxis to a range of chemoattractant substances across fibronectin-coated membranes. In vivo, a decreased neutrophil infiltration in the skin was observed following application of imiquimod to induce psoriasis-like lesions, despite normal epidermal production of chemoattractant by plasmacytoid DCs and keratinocytes. The reduced chemotaxis and emigration of *Stim1*^*−/−*^ neutrophils observed in this study using conditional genetic ablation is thus at odds with the two previous studies using chimeric mice (13, 126).

Subsequently, Sogkas et al. (109) studied the recruitment of neutrophils by the anaphylatoxin C5a in mice transplanted with bone marrow cells from *Stim1*^*−/−*^ and *Orai1*^*−/−*^ mice. *Stim1* and *Orai1* ablation differentially impacted the responses evoked by agonists or by store depletion, with *Stim1* ablation blunting SOCE by ∼70% without impacting agonist-evoked Ca^2+^ elevations, whereas the converse was observed in *Orai1*^*−/−*^ neutrophils. Consistent with the diverging Ca^2+^ phenotypes, chemotaxis triggered by C5a, fMLP, or LPS-primed biological fluids was normal in *Stim1*^*−/−*^ and decreased in *Orai1*^*−/−*^ neutrophils. In vivo, neutrophil recruitment was decreased in *Orai1*^*−/−*^ chimeric mice and slightly increased in *Stim1*^*−/−*^ mice following injection of C5a or LPS into the peritoneal cavity. Neutrophil emigration was reduced by both *Orai1* and *Stim1* deletion in a hypersensitivity pneumonitis model that uses an immune complex-mediated reaction involving the release of chemoattractants by alveolar macrophages, which itself was normal in *Orai1*^*−/−*^ and reduced in *Stim1*^*−/−*^ chimeric mice. These data reveal contrasting roles for ORAI1 and STIM1 in Ca^2+^-dependent neutrophil migration, with ORAI1 required for agonist-mediated Ca^2+^ entry and efficient neutrophil emigration into tissue and STIM1 required for SOCE but dispensable for neutrophils migration.

In a series of elegant papers, the Simon group studied the role of ORAI1 in neutrophil migration under shear flow. In ORAI1-depleted HL-60 cells or neutrophils from heterozygous *Orai1*^*+/−*^ mice, Ca^2+^ elevations associated with the capture of neutrophils on ICAM-1- and E-selectin-coated surfaces, neutrophil arrest, polarization, and directional migration under shear flow were reduced (26, 101). Interestingly, the Ca^2+^ elevations initiated by activation of high-affinity LFA-1 (CD11a/CD18) were more deficient than the response to chemotactic peptides, suggesting that ORAI1 is specifically required for integrin-mediated outside-in signaling. Accordingly, ORAI1 depletion reduced the size of LFA-1 clusters and F-actin polymerization, causing a defect in adhesion strengthening under shear stress. In vivo, the defect in adhesion strengthening translated into reduced neutrophil recruitment in skin wounds (25). These data indicate that ORAI1 meditates local Ca^2+^ signals at sites of high-affinity LFA-1 engagement to sustain adhesion strengthening and the directional migration of neutrophils.

In summary, all studies in mice report alteration in Ca^2+^ signaling in *Stim1*^*−/−*^ neutrophils. The defects range from subtle alterations in local signals generated at the ER-phagosomal interface (73) to defects in global Ca^2+^ signals evoked by receptor ligation and store depletion (109, 112, 126). Two studies report reduced phagocytosis, and one study a reduced production of oxygen radicals, but only one out of four studies reported reduced chemotaxis. This study was the only one to use fibrinogen-coated surfaces, a setting that induces concomitant integrin, selectin, and chemokine receptor activity that possibly accounts for the migration defect observed in vitro. However, this study also reported reduced neutrophil emigration into skin wounds in vivo, while normal emigration of *Stim1*^*−/−*^ neutrophils in the lung and peritoneum was reported in two other studies. Whether *Stim1* ablation impairs neutrophil migration might thus depend on the panel of cytokines released at the infection or inflammation sites and on the type of migration involved, as neutrophils use different mechanisms to reach different tissues (49). In two studies, the cellular defects observed in *Stim1*^*−/−*^ neutrophils correlated with increased bacterial infections and faster recovery from psoriasis-like lesions (112, 126). Regarding ORAI1, three studies from the same group report reduced local Ca^2+^ signals at focal sites of high-affinity integrin engagement (26, 101), while one study reports normal SOCE but reduced responses to chemoattractants in neutrophils from *Orai1*^*−/−*^ chimeric mice. In all of these studies, the Ca^2+^ signaling defects were consistently associated with defective neutrophil migration. STIM1, therefore, appears to mediate local and global Ca^2+^ signals that control the bactericidal capacity of mouse neutrophils and possibly migration into specific tissues, while ORAI1 is involved in integrin-mediated neutrophil guidance and in the migration toward chemoattractants.

### Patients with STIM1 and ORAI1 mutations.

At odds with all of the data obtained in mice and studies in HL-60 cells, a very recent study in patients with loss of function mutations reported normal Ca^2+^ responses and preserved effector functions in ORA1 and STIM1-deficient neutrophils (Table 2) (30). The index patient with the ORAI1 p.R91W loss of function mutation, now 19 yr old, had undergone complete autologous reconstitution of his myeloid and lymphoid compartments and exhibited again a complete lack of SOCE in T lymphocytes. Surprisingly, robust Ca^2+^ entry following activation with fMLP, streptococci, and store depletion was observed in neutrophils obtained from this patient. In keeping with the normal Ca^2+^ signals, normal IL-8 secretion, ROS production, adhesion, chemotaxis, and phagocytosis were observed in the ORAI1-impaired human neutrophils. Nearly identical results were obtained in neutrophils from a patient bearing the STIM1 pR429C mutation, which abolishes SOCE in T cells by interfering with the binding of STIM1 to ORAI1 (65). Again, normal Ca^2+^ responses, together with normal IL-8 secretion, ROS production, static adhesion, and chemotaxis, was observed in the STIM1-deficient neutrophils. These surprising data indicate that the loss of function mutations in STIM1 and ORAI1 that abolish SOCE in T cells have a very marginal effect on SOCE in human neutrophils. Another study also reported normal neutrophil functions in a family bearing the homozygous missense mutation p.L74P in the EF-hand domain of STIM1, which abrogated SOCE and impaired effector functions in natural killer cells without causing overt immunodeficiency (82). Thus, although studies from mice and HL-60 cells point to an important role for STIM1 and ORAI1 in the control of Ca^2+^-dependent neutrophil functions, Ca^2+^ signaling and antibacterial functions of human neutrophils may rely on multiple redundant pathways. Whether this is simply compensated by the other STIM and ORAI isoforms, or whether other Ca^2+^ signaling mechanisms are involved remains to be determined.

## Macrophages

Macrophages, like neutrophils, kill and clear invading pathogens through phagocytosis, via the acidification and release of enzymes within their phagosomes (1). Phagosomal ROS production is lower in macrophages than in neutrophils, while the acidification is more rapid and extensive due to rapid delivery of V-ATPase. Like DCs as well, however, macrophages link the innate and adaptive immune system by presenting antigens to lymphocytes, an event that can eventually lead to the production of antibodies, thereby ensuring lasting and robust immune responses. Macrophage functions extend beyond innate and adaptive immunity. Macrophages additionally promote wound healing and tissue repair, while resident macrophages established inside tissues, such as the lung (alveolar macrophages) and liver (Kupfer cells), maintain tissue homeostasis by clearing dead cells and debris from the organism. They also play less beneficial roles as they can infiltrate tumors and promote tumor growth and progression. According to their functions, macrophages are classified into two main subtypes, the M1 “killer” macrophages classically activated by LPS and interferon-γ that secrete high levels of proinflammatory cytokines like IL-12, and the alternately activated M2 “repair” macrophages producing anti-inflammatory cytokines like IL-10. As the names imply, proinflammatory M1 macrophages kill bacteria and process antigens for presentation, while anti-inflammatory M2 macrophages contribute to tissue repair but also promote tumor growth.

In keeping with the diversity and versatility of macrophages, the evidence linking Ca^2+^ elevations to the control of macrophage functions is disparate and somewhat contradictory. Early studies revealed that receptors for platelet-activating factor and for the chemotactic peptide fMLP induce SOCE, implicating SOCE in the control of macrophage function (3, 48, 57, 93). Accordingly, activation of purinergic receptors by extracellular ATP released by dying cells generates intracellular Ca^2+^ signals in macrophages (21, 116), and these signals contribute to the release of IL-1 by murine peritoneal macrophages (17). The ATP-generated Ca^2+^ elevations were subsequently shown to mediate killing of *Mycobacterium bovis* by promoting macrophage phagosomal acidification (114) and to be required for the induction of the NLRP3 (nucleotide-binding domain, leucine-rich-repeat-containing family, pyrin domain-containing 3) inflammasome in bone marrow-derived macrophages (BMDMs) (69) (reviewed in Ref. 120). Ca^2+^ elevations also occur during FcγR-mediated phagocytosis in macrophages (41, 50, 122) following the clustering of FcγR subtypes, such as hFcγRI and hFcγRIIA that activate PLCγ1 and -γ2 (60, 94), but these signals are dispensable for F-actin assembly and disassembly during the phagocytic process (39). Phagocytosis can proceed normally in macrophages upon intracellular Ca^2+^ chelation (24, 41, 66), unless it is specifically mediated by the hFcγRIIA receptor (79), suggesting that some receptors signal preferentially via Ca^2+^, but that this restriction can be bypassed when multiple receptors are engaged. In contrast to neutrophils, cytosolic Ca^2+^ elevations appear less stringently required for the maturation of phagosomes, such as phagolysosome fusion, and the activation of the superoxide-generating NADPH oxidase complex (70, 130) (reviewed in Ref. 75).

Based on these premises, the diverging outcome of altered STIM1-ORAI coupling on macrophage functions in animal models is not too surprising (Table 3). The first study with chimeric bone marrow *Stim1*^*−/−*^ mice reported nearly abrogated SOCE and markedly reduced Ca^2+^ elevations upon FcγR cross-linking in peritoneal macrophages (13). In addition, intracellular Ca^2+^ store content was reduced by 50%, and peritoneal macrophages with altered FcγR isotype distribution were observed. Strikingly, FcγR-dependent phagocytosis of IgG-opsonized red blood cells was nearly abrogated, while the production of the monocyte chemotactic protein-1 was preserved. Reduced phagocytosis was also observed in Kupfer cells upon induction of IgG-dependent autoimmune hemolytic anemia (AIHA), while C5a bioactivity and cytokine secretion in the alveolar fluid were blunted in a model of hypersensitivity pneumonitis. Consequently, chimeric *Stim1*^*−/−*^ mice were protected against AIHA as well as autoimmune thrombocytopenia and anaphylaxis induced by the injection of anti-platelet antibodies, an IgG-dependent response mediated by macrophages and mast cells. This study thus establishes STIM1 as an essential mediator of autoimmune inflammation that controls FcγR-mediated signaling in macrophages.

In a follow-up study, Sogkas et al. (108) used these chimeric *Stim1*^*−/−*^ mice together with global and chimeric *Stim2*^*−/−*^ mice to study the contributions of STIM1 and STIM2 in macrophage functions. SOCE and store content were decreased to a similar extent in peritoneal macrophages isolated from chimeric *Stim1*^*−/−*^ and global *Stim2*^*−/−*^ mice, but the Ca^2+^ elevations evoked by cross-linking of FcγRIII and FcγRIV were only marginally decreased in *Stim2*^*−/−*^ cells. This milder Ca^2+^ signaling defect correlated with a milder inhibition of phagocytosis that could be rescued by the addition of C5a or of liver supernatant from autoimmune mice to *Stim2*^*−/−*^ macrophages. Unexpectedly and contrary to the mild Ca^2+^ phenotype, CCL2 and C5a-induced chemotaxis, as well as Toll-like receptor-4-mediated cytokine release were decreased in *Stim2*^*−/−*^ and normal in *Stim1*^*−/−*^ macrophages. Similar diverging cellular defects were observed in the RAW 264.7 macrophage cell line, where STIM1 silencing inhibited phagocytosis, while STIM2 but not STIM1 silencing reduced chemotaxis (Table 4). Reduced macrophage recruitment and cytokine secretion were also observed following injection of thioglycollate into the peritoneum of global or chimeric *Stim2*^*−/−*^ mice. These migration defects correlated with a reduced production of inflammatory mediators and with increased survival during LPS-induced sepsis in *Stim2*^*−/−*^ mice, which, however, were not protected from AIHA. These data indicate that the two STIM proteins contribute differentially to the control of macrophage effector functions, with FcγR-dependent phagocytosis relying more on STIM1 than on STIM2, whereas chemotaxis and Toll-like receptor-4-induced cytokine release are modulated by STIM2 but not by STIM1.

These conclusions were challenged by a recent study by Vaeth et al. (117), who used inbred C57BL/6 mice with a conditional deletion of the two *Stim* genes (*Stim1*^*fl/fl*^; *Stim2*^*fl/fl*^) under the control of the inducible Mx1-Cre or myeloid-specific Vav-Cre promotors to test the functional impact of SOCE in myeloid cells. Consistent with earlier studies in chimeric mice, SOCE evoked by store depletion or by FcγRII/III cross-linking was nearly absent in BMDMs from these mice. However, ablation of the two STIM proteins did not impact the intracellular Ca^2+^ store content or the differentiation and maturation of BMDMs. Despite the complete lack of SOCE, FcγR-independent phagocytic uptake of nonopsonized latex beads and bacterial phagocytosis of *S. aureus* were normal. Surprisingly, and in complete disagreement with the studies in chimeric mice, FcγR-dependent phagocytosis of opsonized particles and of red blood cells was also completely normal in BMDM and peritoneal macrophages from all of the STIM-deficient mice tested measured at different times and at different phagocytic indexes. In contrast, chelation of cytosolic Ca^2+^ with BAPTA-AM significantly reduced FcγR-dependent phagocytic uptake to a greater extent than nonopsonized targets, demonstrating that intracellular Ca^2+^ elevations positively regulate phagocytosis by macrophages. Phagosomal maturation was equally unaffected as lysosomal marker recruitment to phagosomes was comparable to wild-type mice. The authors then tested other macrophage functions and observed normal production of a battery of cytokines (IL-2, IL-6, IL-10, IL-12/23p40, IL-12p70, TNF-α) in BMDM exposed to synthetic or natural ligands of PRRs known to mediate Ca^2+^ signals (LPS, curdlan, Bacillus Calmette-Guérin), indicating that STIM proteins and SOCE are dispensable for cytokine production by macrophages. Finally, IL-1β secretion, cleavage of procaspase 1 into active caspase 1, and intracellular levels of pro-IL-1β were normal in BMDMs from *Stim1*^*fl/fl*^*Mx1-Cre* mice and *Stim1*^*fl/fl*^; *Stim2*^*fl/fl*^ *Vav-Cre* mice stimulated with LPS and ATP, and with monosodium urate crystals or bacterial flagellar toxin to induce the activation of the NLRP3 and NLRC4 inflammasome, respectively. Overall, this comprehensive study clearly demonstrates that STIM1 and STIM2 proteins are not required for phagocytosis, cytokine secretion, or inflammasome activation in macrophages.

Whether the differences in findings obtained with inbred mice with conditional myeloid ablation of the two *Stim* genes and radiation chimeric mice reconstituted with bone marrow cells from *Stim1*^*−/−*^ mice result from differences in the genetic background of the mice used, or whether added radiation-induced stress can account for these differences remains to be determined, but it is conceivable that the additional defects in ER Ca^2+^ content may contribute in part to the discrepancies. Future studies confirming or disproving the differential effects of STIM2 ablation, or the effects of STIM protein on migration, will also be informative in discerning how Ca^2+^-dependent functions are regulated in macrophages.

## DCs

DCs are phagocytic immune cells whose fundamental role is to engage and instruct the adaptive immune system. It is generally believed that, unlike neutrophils and to a certain extent macrophages, the function of DCs is not to kill ingested particles. Instead they are specialized to integrate and use the ingested material as well as the signals received during the ingestion encounter to then present antigens to naive T cells, and, depending on the context, help initiate T-helper, cytotoxic, or immunosuppressive responses (7, 37, 80). Most DCs are short-lived and are continually replaced by differentiation of myeloid hematopoietic precursors circulating in the blood. DCs are heterogeneous, and at least seven different subtypes have been identified that show differences in cell surface marker expression as well as function, although all are thought to originate from a common hematopoietic precursor (67). Under steady state, DCs constantly survey tissues and then migrate to the spleen and lymph nodes where they interact with and present antigens to T cells and either die or reenter the circulation (7, 67). Similar to neutrophils and macrophages, DCs respond to PAMPs and DAMPs via PRRs (118). PRR activation can also induce the differentiation of precursors into immature DCs or a further maturation of immature DCs. Mature DCs increase their migratory behavior and express a different set of cell surface molecules and secrete different cytokines, all of which influence the manner by which DCs interact with their environment (7, 37, 118). In addition, DC maturation affects the way that phagocytosed material is processed, for example by increasing phagosomal ROS production and decreasing phagosomal acidification, events that activate different subsets of proteolytic enzymes within phagosomes and determine the efficiency and types of molecules that will be loaded onto major histocompatibility complex (MHC) molecules (99). Together, all of the different functions that are modified by DC maturation will determine the immune outcome of antigen-dependent DC-T-cell interactions.

Pharmacological manipulations have linked Ca^2+^ signaling to multiple DC functions, including cell-surface marker upregulation accompanying differentiation and maturation, phagocytosis, cytokine secretion, migration, and antigen presentation (reviewed in Refs. 19, 104). Similar to macrophages, the earlier studies on the Ca^2+^ function in DCs suffered from a number of inconsistencies, which may partly arise from the use on nonspecific pharmacology, differences in cellular models, as well as the phenotypic plasticity of DCs due to their heterogeneity or state of maturation. Interestingly, in an early study, the group of Clapham recorded *I*_crac_ currents in response to ER Ca^2+^ depletion or stimulation with ATP, but not voltage-dependent currents in patch-clamped bone marrow derived mouse DCs (BMDCs), and made the provocative suggestion that “pure” (what is now defined as STIM-ORAI mediated) SOCE may be the only major Ca^2+^ entry pathway in DCs (44), thus raising the question of the exact role of STIM and ORAI proteins in DC function.

Although STIM1 and ORAI1-3 transcripts were detected in mouse and human DCs in previous studies (45, 64), the first report to show STIM and ORAI expression at the protein level in DCs was that of Bandyopadhyay and colleagues (8). Here, the authors examined STIM1-2 and ORAI1-3 expression in mouse BMDCs by Western blotting, and, using expression in T cells as a benchmark, observed that STIM2 and ORAI2 expression was much higher in DCs. Using immunofluorescence and immunoprecipitation, translocation and interaction of STIM2 and ORAI2, but not STIM1 and ORAI1, were observed upon store depletion, although the specificities of the antibodies were not tested. Based on these data, the authors concluded that STIM1 and ORAI1 were not involved in DC SOCE. However, a few subsequent studies hinted to the contrary. First, one study showed in human DCs increased STIM1 and ORAI1 protein and mRNA expression in response to LPS, a maturation-inducing PAMP shown to elicit *I*_crac_ currents in DCs (64), that imparts BAPTA- and xestospongin C-sensitive increases in cell surface maturation markers (6), as well as Ca^2+^- and nuclear factor of activated T cell-mediated IL-2 production (124) in mouse (64, 124) and human (6) DCs. Second, a series of studies from the group of Lang showed that differences in fura-2 measured SOCE or *I*_crac_ current densities observed in *Ampk*^−/−^ (76), *Sgk*^−/−^ (102), or mutant glycogen synthase kinase knock-in mouse BMDCs (103) correlated with STIM1, STIM2, and ORAI1 protein levels, as well as LPS-induced cell surface markers and CXCL12-induced migration.

The first reported genetic manipulation of STIM1 and ORAI1 in DCs was published by Felix and colleagues (31) in 2013. In this report, transfection of human DCs with small interfering RNA directed against either STIM1 or ORAI1 reduced SOCE by ∼60%, as well as thapsigargin-, LPS-, zymosan- and TNFα-induced upregulation of maturation markers (Table 5), although no quantification or loading controls were provided to judge knock-down efficiency. Interestingly, STIM2 transcripts were not detected in human DCs (31). Subsequently, the first genetic manipulation of STIM proteins in mouse DCs was reported in 2015 by Vaeth et al. (117), where BMDCs from mice with a conditional myeloid ablation of STIM1 and STIM2 were extensively characterized. In BMDCs derived from *Stim1*^*fl/fl*^; *Stim2*^*fl/fl*^ *Vav-Cre* mice, SOCE was completely abrogated, although ER Ca^2+^ stores were not depleted under steady-state conditions. Despite this large defect, surprisingly no functional effects were found. Differentiation from myeloid precursors was normal, or even slightly increased, as judged by the expression of CD11c, CD86, and MHC-CII. Concordantly, conventional and plasmacytoid DC frequencies were similar between a control and the blood of a 5-mo-old patient with an ORAI1 p.R91W homozygous loss-of-function mutation. Upregulation of cell surface maturation markers CD86 and MHC-CII in response to stimulation of various PRR agonists, including LPS, curdlan, imiquimod, CpG, or zymosan, was unchanged as was the secretion of TNF-a, IL-6, IL-10, IL-12p70, IL-23p19, and surprisingly even IL-2, although the large variation as judged by large error bars may have masked significance of smaller decreases or increases, for example in IL-2 in response to CpG or IL-10 in response to curdlan, which appear to be 30% lower or 40% higher, respectively. Similarly, phagocytosis, NLRP3, and NLRC4 inflammasome activation were unchanged. Finally, antigen presentation of soluble ovalbumin to ovalbumin-reactive transgenic CD4^+^ T cells (OT-II cells) was either unchanged or even slightly improved, as judged by T-cell proliferation measured by carboxyfluorescein succinimydil ester dilution or by the higher numbers of IFN-γ-positive T cells. In contrast, phagocytosis, inflammasome activation, and antigen presentation were inhibited by intracellular Ca^2+^ chelation. Thus, while human DCs may rely largely on STIM1 and ORAI1-dependent Ca^2+^ signaling for some PRR-dependent responses, alternative Ca^2+^ signaling pathways may allow mouse DCs to function independently of STIM proteins. Currently, several questions still remain. For instance, as both STIM proteins were ablated in the Vaeth et al. (117) study, whether STIM2 is really the main isoform regulating SOCE in mouse DCs as is the case in neurons (10) and whether it plays a role at all in human DCs remains to be determined. More importantly, it will be interesting to determine whether the discrepancy between the reported function of STIM proteins in human and mouse DCs on maturation markers represents true species-specific differences, or whether experimental conditions can account for the differences observed. Whether other Ca^2+^-dependent DC functions such as migration and phagosomal maturation require SOCE, and whether STIM protein ablation affects DC functions in vivo, also remains unknown.

**Table 5.** Dendritic cell defects in human primary cells and in the mouse model of STIM and ORAI deficiency

Gene	Manipulation	Ca^2+^ Signaling	Maturation/Differentiation	Cytokine Production	Phagocytosis	Ag Presentation	Ref. No.
Human dendritic cells							
*STIM1*	siStim1, human blood-derived DC	Decreased 60% (Tg)	Decreased 30–50% (CD80, CD86, CD83, MHC-CII, in response to Tg, LPS, zymosan, TNF-α)				31
*ORAI1*	siOrai1, human blood-derived DC	Decreased 60% (Tg)	Decreased 30–50% (CD80, CD86, CD83, MHC-CII, in response to Tg, LPS, zymosan, TNF-α)				31

Gene	Mouse Model	Ca^2+^ Signaling	Maturation/Differentiation	Cytokine Production	Phagocytosis	Ag Presentation	Ref. No.
Mouse dendritic cells							
*Stim1* and *Stim2*	Vav-Cre *Stim1*^*−/−*^; *Stim2*^*−/−*^ Myeloid ablation	Decreased >95% (Tg) Normal store content 20% decrease (ATP)	Normal (CD86, MHC-CII, in response to LPS, CpG, zymosan, curdlan, imiquimod)	Normal (IL-2, IL-6, IL-10, IL-12p40, IL-12p70, IL-23p19, TNF-α in response to LPS, CpG, zymosan, curdlan, imiquimod, IL-1b in response to ATP, FlaTox, MSU)	Normal (1:1, 1:5,1:25 unopsonized and IgG-coated beads)	Normal (OT-II CD4+ T cells co-cultured with BMDCs exposed to 500 μg/ml OVA)	117

The table lists the in vitro dendritic cell defects reported in human dendritic cells from peripheral blood and in the mouse model of *Stim1* and *Stim2* deficiency. BMDC, bone-marrow derived dendritic cell; OVA, ovalbumin.

## Concluding Remarks

The accumulation of data over the past 30 yr clearly suggests that a large number of phagocyte functions are governed by Ca^2+^ signals, and early electrophysiological evidence has pointed to SOCE as the major mechanism through which neutrophils, macrophages, and DCs generate intracellular Ca^2+^ signals. Since the discovery of STIM and ORAI proteins as the major molecular players underlying SOCE, however, the subsequent body of literature reported surprisingly mild defects upon STIM and ORAI depletion compared with nonspecific manipulations, such as Ca^2+^ chelation. This implies that, unlike T cells where abrogation of SOCE leads to severe defects in Ca^2+^ dependent functions, phagocytes are likely able to generate intracellular Ca^2+^ signals by alternative means. Since phagocytes are first-line defenders against pathogens, this versatility may have evolved from selective pressure by intracellular pathogens, such as *Mycobacterium tuberculosis*, *Leishmania*, and *Franciscella*, which block Ca^2+^ signals that normally occur during phagocytosis.

Several candidates might compensate the lack of STIM and ORAI as phagocytes possess a large variety of Ca^2+^ signaling molecules at the ER, in acidic Ca^2+^ stores, and at the PM. Expression or function of L-type channels and ryanodine receptors has been documented in neutrophils, macrophages (i.e., Refs. 5, 43, 97, 105, and reviewed in Ref. 20) and DCs (78, 89, 119), although these channels do not appear to mediate Ca^2+^ entry in response to membrane depolarization but to regulate Ca^2+^ release from the ER. Recruitment of Ca^2+^ stores near Ca^2+^ effector proteins can compensate the signaling defects caused by the loss of Ca^2+^ entry channels, as recently shown for junctate in STIM1-deficient phagocytic mouse embryonic fibroblasts (40). Alternatively, Ca^2+^ release from acidic stores may be able to generate local signals, as suggested by studies on TRPM2 knockouts and two-pore channel agonists in DCs (83, 115), neutrophils (54, 110), and macrophages (98). Acidic stores might provide a means of transferring Ca^2+^ to the ER to ensure ER refilling in the absence of STIM-mediated SOCE. Finally, nonselective Ca^2+^-permeable channels of the TRP family, such as TRPM2 and TRPV2 channels, are expressed in innate and adaptive immune cells and have been shown to regulate macrophage chemotaxis and phagocytosis (61, 71, 95) (reviewed in Ref. 34).

In summary, more research is still needed to define the precise role of STIM and ORAI isoforms in the function of phagocytic cells. This is important for both fundamental research and clinical applications. The identification of molecules in the Ca^2+^ toolkit of each phagocytic cell type will provide fundamental knowledge on the mechanisms governing Ca^2+^-dependent phagocyte functions. This knowledge is needed to allow the manipulation of defined targets by genetic and pharmacological means for therapeutic strategies. Specific inhibitors or activators of STIM and ORAI are already being developed and are suggested to be useful for treating bacterial infections and autoimmune diseases (2, 23). A better knowledge of STIM and ORAI function in phagocytes is needed to evaluate the immunosuppressive danger such strategies might hold and to identify potentially safer isoform-specific manipulations.

## DISCLOSURES

No conflicts of interest, financial or otherwise, are declared by the author(s).

## AUTHOR CONTRIBUTIONS

Author contributions: N.D. and P.N. drafted manuscript; N.D. and P.N. edited and revised manuscript; N.D. and P.N. approved final version of manuscript.
